# Supplementary material for: Identification of a Novel IncHI1B Plasmid in MDR Klebsiella pneumoniae 200 from Swine in China
Source: Antibiotics (Basel). 2022 Sep 9;11(9):1225. doi: 10.3390/antibiotics11091225 (PMC9494989; doi:10.3390/antibiotics11091225)
Supplement: Supplementary file 1 [file antibiotics-11-01225-s001.zip › Figure S1.pdf]

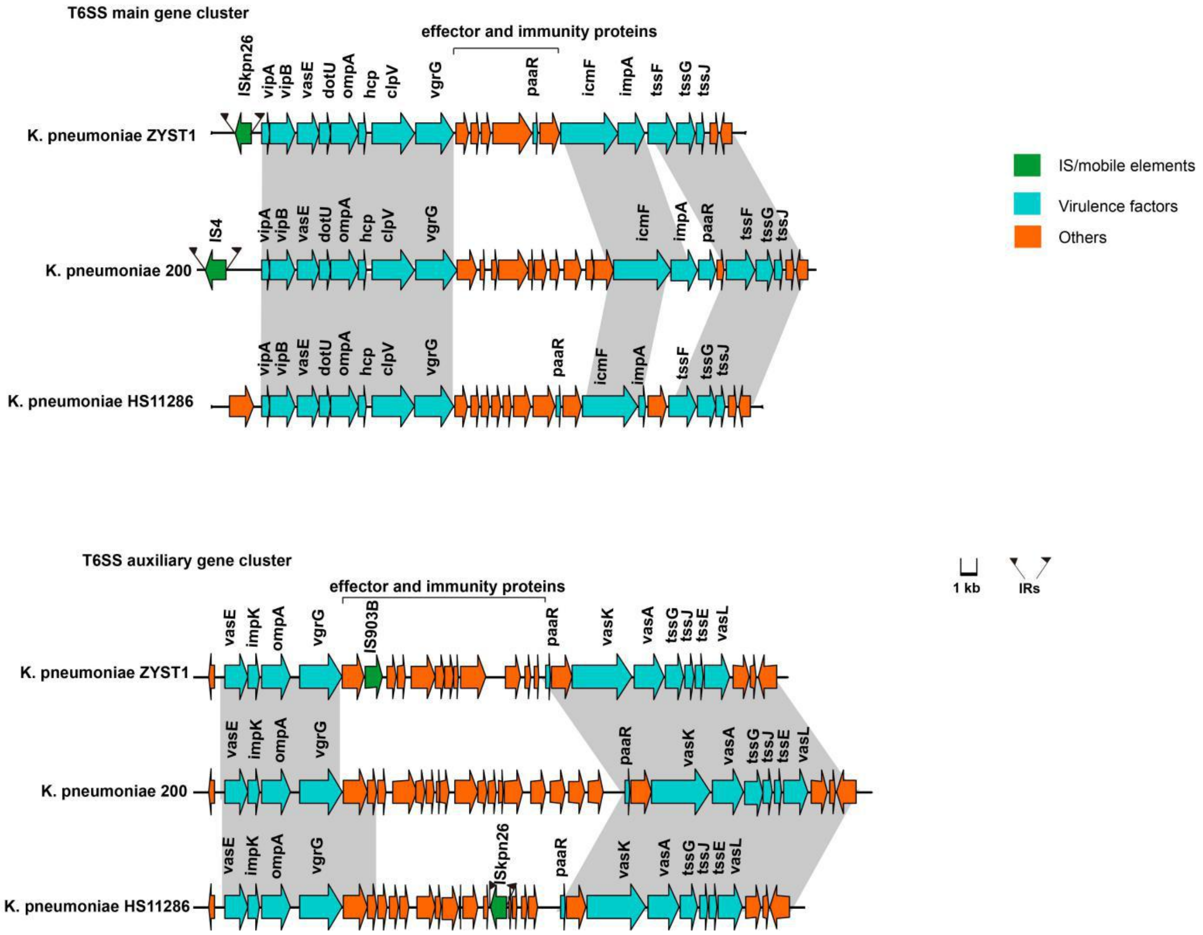

Figure S1. linear illustration of the T6SS gene clusters of 200 and comparative analysis of this region with that of *K. pneumoniae* HS11286 and *K. pneumoniae* ZYST1. To facilitate comparison, the sequence is shown according to the orientation described for the T6SS gene clusters of *K. pneumoniae* HS11286. Genes are represented and classified by function into different groups with different colors. ISs are represented by arrows, showing the direction of transcription of the transposase genes. Flags represent the IRs of ISs.
