# Supplementary material for: Identification of a Novel IncHI1B Plasmid in MDR Klebsiella pneumoniae 200 from Swine in China
Source: Antibiotics (Basel). 2022 Sep 9;11(9):1225. doi: 10.3390/antibiotics11091225 (PMC9494989; doi:10.3390/antibiotics11091225)
Supplement: Supplementary file 1 [file antibiotics-11-01225-s001.zip › Figure S3.pdf]

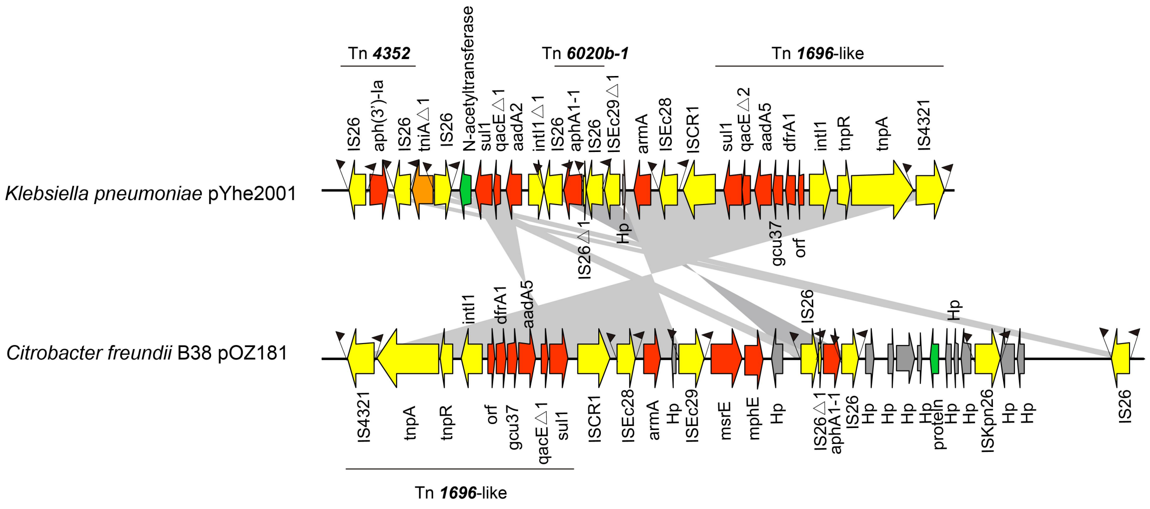

Figure S3 Linear illustration of the MDR region of plasmid pYhe2001 and comparative analysis of this region in pOZ181 from *Citrobactere freundii* B38. ORFs are shown as arrows, indicating the transcription direction, and the colors of the arrows represent different fragments. Intact ISs are represented by arrows, showing the direction of transcription of the transposase genes. Flags represent the IRs of ISs and transposons. Homologous gene clusters in different isolates are shaded in gray (>97%).
