# Supplementary material for: Identification of a Novel IncHI1B Plasmid in MDR Klebsiella pneumoniae 200 from Swine in China
Source: Antibiotics (Basel). 2022 Sep 9;11(9):1225. doi: 10.3390/antibiotics11091225 (PMC9494989; doi:10.3390/antibiotics11091225)
Supplement: Supplementary file 1 [file antibiotics-11-01225-s001.zip › Table S1.pdf]

Table S1. Antimicrobial resistance profile of *K. pneumoniae* 200

Antimicrobial agents	Profile
Cefotaxime	R
Ceftazidime	R
Cefoxitin	R
Oxacillin	R
Meropenem	S
Imipenem	S
amikacin	R
Gentamicin	R
Kanamycin	R
Streptomycin	R
Ciprofloxacin	R
Chloramphenicol	R
Erythromycin	R
Tetracycline	R
Trimethoprim-Sulfamethoxazole	R
