# Supplementary material for: Identification of a Novel IncHI1B Plasmid in MDR Klebsiella pneumoniae 200 from Swine in China
Source: Antibiotics (Basel). 2022 Sep 9;11(9):1225. doi: 10.3390/antibiotics11091225 (PMC9494989; doi:10.3390/antibiotics11091225)
Supplement: Supplementary file 1 [file antibiotics-11-01225-s001.zip › Table S2.pdf]

Table S2. Characteristics of *K. pneumonia* strain 200

Parameter	Chromosome
Size(bp)	5,257,665
G+C(%)	58.78
No. of predicted coding sequences	5,106
Resistance genes	*gyrB*, *emrD*, *eefC*, *eefB*, *eefA*, *acrF*, *acrE*, *acrS*, *folP*, *evgA*, *tolC*, *parE*, *basR*, *parC*, *mdtP*, *mdtO*, *mdtN*, *dfrE*, *emrB*, *emrA*, *oqxB*, *oqxA*, *acrD*, *gyrA*, *baeR*, *baeS*, *mdtD*, *mdtC*, *mdtB*, *mdtA*, *ompC*, *sdiA*, *hns*, *mdtM*, *adeR*, *kexd*, *mdtK*, *katG*, *marR*, *marA*, *bla*$_{SHV-182}$, *phoP*, *phoQ*, *mdtH*, *mdtG*, *macB*, *macA*, *mdeA*, *eptA*, *PmrA*, *PmrB*, *ramA*, *acrR*, *acrA*, *acrB*, *mdtG*, *phoE*, *folA*, *robA*, *mdtM*, *fosA5*, *soxR*, *soxS*, *rpoC*, *rpoB*, *cpxR*, *cpxA*, *mdtD*, *mdtL*, *sul*, *qacE*
Virulence genes	*fepC*, *msbA*, *ilpA*, *rfaF*, *rfaD*, *entA*, *manB*, *gmhA*, *ureG*, *ureB*, *rfaE*, *fimE*, *fimB*, *fimF*, *fimH*, *focA*, *fimA*, *fimD*, *sfaF*, *focD*, *fimC*, *entE*, *entF*, *shuU*, *fepD*, *fepG*, *chuS*, *shuS*, *fepA*, *algU*, *entC*, *iroN*, *iroE*, *iutA*, *galU*, *rffG*, *entB*, *kdsA*, *fepB*, *sodB*, *mgtC*, *acpXL*, *kdsB*, *entS*, *fes*, *clpP*, *ecpB*, *ecpD*, *ecpC*, *ecpA*, *ecpR*, *lpxB*, *lpxA*, *lpxD*, *htpB*, *icl*, *mrkA*, *mrkB*, *mrkC*, *mrkD*, *mrkE*, *mrkF*, *mrkJ*, *mrkI*, *mrkH*
