# Supplementary material for: Identification of a Novel IncHI1B Plasmid in MDR Klebsiella pneumoniae 200 from Swine in China
Source: Antibiotics (Basel). 2022 Sep 9;11(9):1225. doi: 10.3390/antibiotics11091225 (PMC9494989; doi:10.3390/antibiotics11091225)
Supplement: Supplementary file 1 [file Figure S2.pdf › Figure S2.pdf]

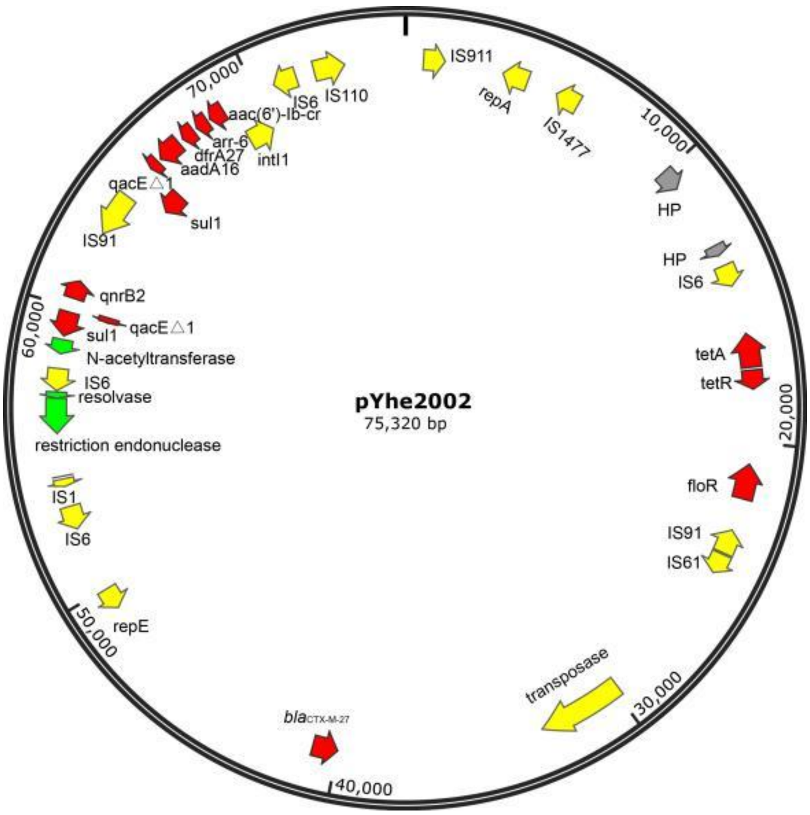

Figure S2 Circular representation of plasmid pYhe2002. Genes for IS/mobile element 13 (yellow), hypothetical protein (gray), antibiotic resistance (red), and other (green) are 14 depicted.
